# Left ventricular blood flow kinetic energy after myocardial infarction - insights from 4D flow cardiovascular magnetic resonance

**DOI:** 10.1186/s12968-018-0483-6

**Published:** 2018-08-30

**Authors:** Pankaj Garg, Saul Crandon, Peter P. Swoboda, Graham J. Fent, James R. J. Foley, Pei G. Chew, Louise A. E. Brown, Sethumadhavan Vijayan, Mariëlla E. C. J. Hassell, Robin Nijveldt, Malenka Bissell, Mohammed S. M. Elbaz, Abdallah Al-Mohammad, Jos J. M. Westenberg, John P. Greenwood, Rob J. van der Geest, Sven Plein, Erica Dall’Armellina

**Affiliations:** 1 Leeds Institute of Cardiovascular and Metabolic Medicine (LICAMM), University of Leeds, Leeds, LS2 9JT UK; 2 Department of Cardiology, Academic Medical Center, University of Amsterdam, Amsterdam, The Netherlands; 3 Department of Radiology, Leiden University Medical Center, Leiden, The Netherlands; 4 Sheffield Teaching Hospitals NHS Foundation Trust, Sheffield, UK

**Keywords:** 4D flow CMR, Myocardial infarction, Hemodynamics, Flow quantification

## Abstract

**Background:**

Myocardial infarction (MI) leads to complex changes in left ventricular (LV) haemodynamics that are linked to clinical outcomes. We hypothesize that LV blood flow kinetic energy (KE) is altered in MI and is associated with LV function and infarct characteristics. This study aimed to investigate the intra-cavity LV blood flow KE in controls and MI patients, using cardiovascular magnetic resonance (CMR) four-dimensional (4D) flow assessment.

**Methods:**

Forty-eight patients with MI (acute-22; chronic-26) and 20 age/gender-matched healthy controls underwent CMR which included cines and whole-heart 4D flow. Patients also received late gadolinium enhancement imaging for infarct assessment. LV blood flow KE parameters were indexed to LV end-diastolic volume and include: averaged LV, minimal, systolic, diastolic, peak E-wave and peak A-wave KEi_EDV_. In addition, we investigated the in-plane proportion of LV KE (%) and the time difference (TD) to peak E-wave KE propagation from base to mid-ventricle was computed. Association of LV blood flow KE parameters to LV function and infarct size were investigated in all groups.

**Results:**

LV KEi_EDV_ was higher in controls than in MI patients (8.5 ± 3 μJ/ml versus 6.5 ± 3 μJ/ml, *P* = 0.02). Additionally, systolic, minimal and diastolic peak E-wave KEi_EDV_ were lower in MI (*P* < 0.05). In logistic-regression analysis, systolic KEi_EDV_ (Beta = − 0.24, *P* < 0.01) demonstrated the strongest association with the presence of MI. In multiple-regression analysis, infarct size was most strongly associated with in-plane KE (*r* = 0.5, Beta = 1.1, *P* < 0.01). In patients with preserved LV ejection fraction (EF), minimal and in-plane KEi_EDV_ were reduced (*P* < 0.05) and time difference to peak E-wave KE propagation during diastole increased (*P* < 0.05) when compared to controls with normal EF.

**Conclusions:**

Reduction in LV systolic function results in reduction in systolic flow KEi_EDV_. Infarct size is independently associated with the proportion of in-plane LV KE. Degree of LV impairment is associated with TD of peak E-wave KE. In patient with preserved EF post MI, LV blood flow KE mapping demonstrated significant changes in the in-plane KE, the minimal KEi_EDV_ and the TD. These three blood flow KE parameters may offer novel methods to identify and describe this patient population.

**Electronic supplementary material:**

The online version of this article (10.1186/s12968-018-0483-6) contains supplementary material, which is available to authorized users.

## Background

Myocardial infarction (MI) is one of the leading causes of death and disability worldwide. The effects of acute MI on left ventricular (LV) haemodynamics [1] and the prognostic importance of infarct size and LV ejection fraction (EF) following MI are well established [2]. Large infarcts are associated with an increased LV wall stress secondary to increased pre-load on the LV [3, 4]. This and subsequent adverse effects on LV afterload play a crucial role in infarct expansion and dilatation leading to adverse LV remodelling [5]. The assessment of LV haemodynamics and flow provides incremental prognostic evaluation in patients with MI [6]. Two-dimensional (2D) Doppler echocardiography is the mainstay for the non-invasive assessment of LV haemodynamics. However, Doppler echocardiography provides unidirectional velocity information and does not inform about three-dimensional intra-cavity flow [7].

**Fig. 1:**
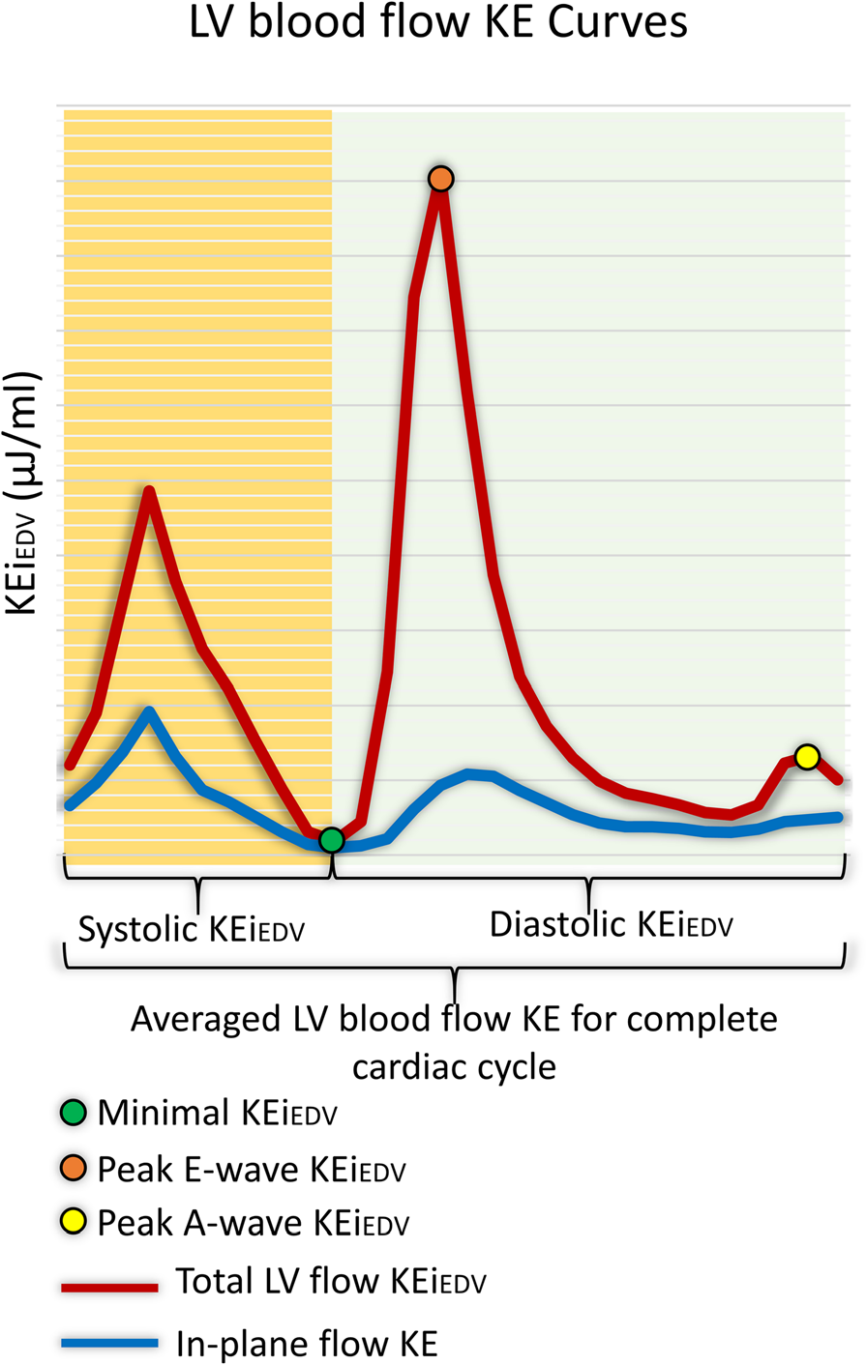
Illustration of all the left ventricular blood flow kinetic energy (KE) parameters investigated in this study in a healthy control

The kinetic energy (KE) of the blood represents a fundamental component of work performed by the heart which results in the movement of the blood [8]. Four-dimensional flow (4D flow) cardiovascular magnetic resonance (CMR) imaging allows semi-automatic quantification of intra-cavity LV flow KE parameters in three dimensions (3D) [9–15]. This further allows to compute several parameters of KE at different time points in the cardiac cycle (Fig. 1). Previous studies have demonstrated LV systolic average KE is higher in patients with heart failure compared to healthy controls, but lower when indexing to heart size [16]. Another study has shown that the KE of the portion of flow that passes directly through the LV in a single cardiac cycle or ‘direct flow’ diminishes with progressive LV dilatation [17]. Recently, Wong et al. demonstrated that in healthy individuals, early peak diastolic KE progressively decreases with age, whereas systolic peaks remain constant [18]. The effects of MI on 3D LV blood flow KE have been described by Kanski et al. in a study which recruited MI patients as a sub-group [16]. However, more focused validation studies with larger MI patient population are needed to investigate if there is association of mechanical function, infarct characteristics and LV blood flow KE. It also remains unclear if significant differences in LV KE are seen in acute versus chronic MI.

Hence, the aims of this study were as follows: a) quantify dynamic parameters of LV blood flow KE in patients with acute and chronic MI and age and gender matched healthy controls; b) investigate the reproducibility of LV blood flow KE parameters; c) investigate if there are significant changes in LV blood flow KE in different sub-groups of left-ventricular ejection fraction; d) investigate the association of LV KE to infarct size and if LV KE is altered in acute versus chronic MI patients.

## Methods

### Study population

**Fig. 2:**
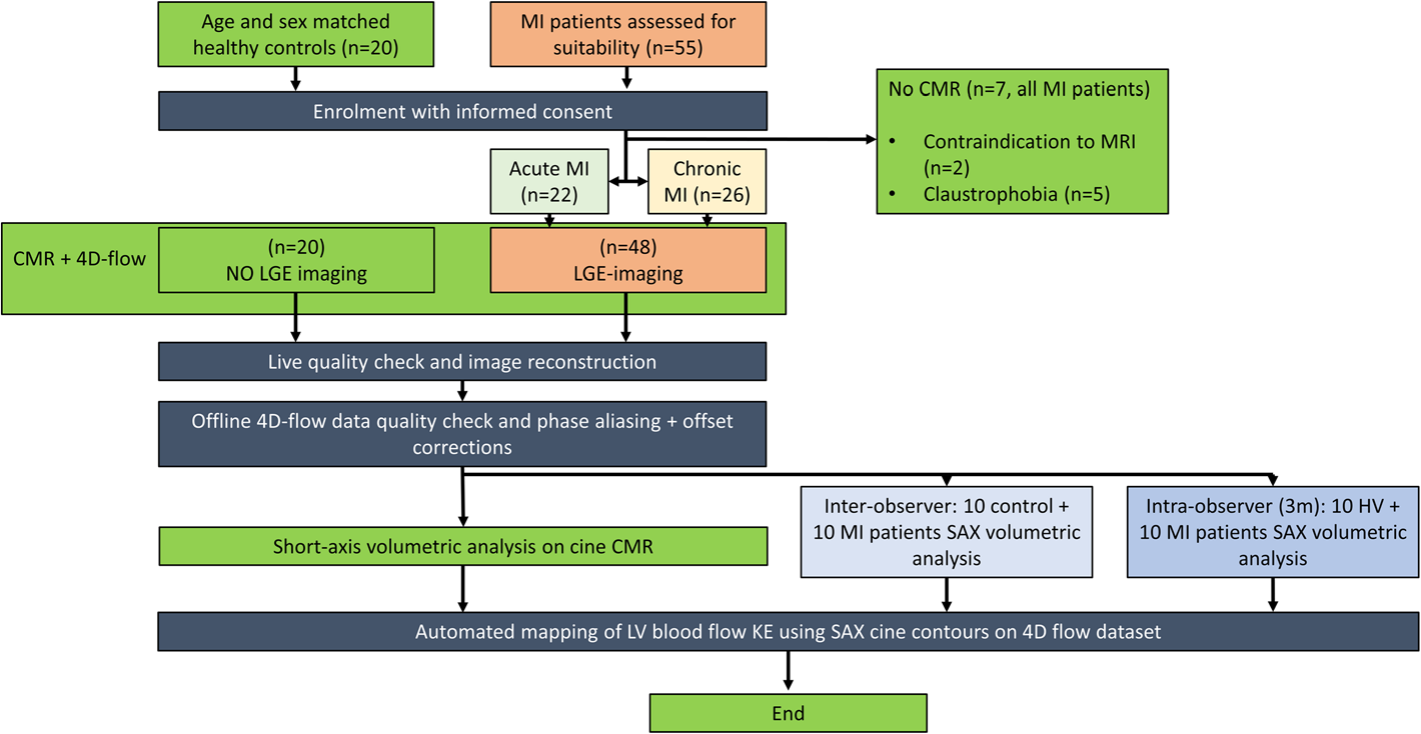
Study design. 4D = four-dimensional, 4DF = four-dimensional flow, CMR = cardiovascular magnetic resonance imaging, HV = healthy volunteers, KE = kinetic energy, LGE = late gadolinium enhancement imaging, LV = left ventricle, m = month, MI = myocardial infarction, CMR = cardiovascular magnetic resonance and SAX = short-axis

The study design is detailed in Fig. 2. Patients with acute MI patients were prospectively identified following an admission with ST-elevation myocardial infarction (STEMI) at Leeds. Patients with chronic MI were prospectively identified from the cardiology outpatient clinics in Leeds. Age and gender matched healthy controls were prospectively recruited from two centres: Leeds, UK and Leiden, The Netherlands.

The inclusion criteria for patients with acute MI were: patients with first time acute ST elevation MI (STEMI) treated by primary percutaneous coronary intervention (PPCI) within 12-h of onset of chest pain. Acute STEMI was defined as per the current international guidelines [19]. Acute STEMI patients were scheduled for CMR imaging within 72-h of indexed presentation. The inclusion criteria for patients with chronic MI were: previous history of MI and the presence of ischaemic scar on late gadolinium enhancement (LGE) imaging. Exclusion criteria for all patients included: non-ischaemic cardiomyopathy, severe valvular heart disease, estimated glomerular filtration rate < 30 ml/min/1.73m^2^, haemodynamic instability (Killip class III/IV requiring on-going intravenous diuretic therapy [20]) and contraindications to CMR imaging.

Healthy control subjects had no history or symptoms of cardiovascular disease, were not on cardiovascular or other relevant medication and had no contraindications to CMR.

### CMR examination

All healthy control subjects and patients underwent CMR imaging on identical 1.5 T systems at both sites (Ingenia, Philips Healthcare, Best, The Netherlands); with a 28-channel flexible torso coil and digitization of the CMR signal in the receiver coil.

### CMR protocol and image acquisition

The CMR protocol was as follows:

1. Survey images
2. The following cines were defined using survey: vertical long-axis, horizontal long-axis, 3-chamber (LVoutflow tract-views), and the LV volume contiguous short axis stack. All cines were acquired with a balanced steady-state free precession (bSSFP), single-slice breath-hold sequence. Typical parameters for bSSFP cine were as follows: SENSE factor 2, flip angle 60°, echo time (TE) 1.5 milliseconds, repetition time (TR) 3 milliseconds, field of view (FOV) 320–420 mm according to patient size, slice thickness 8 mm, and 30 phases per cardiac cycle.
3. Contrast injection (Magnevist: 0.2 mmol/kg) [only done in the patient population]
4. For whole heart 4D flow, field of view (FOV) was planned in trans-axial plane making sure whole heart was in FOV. 4D flow was done using fast field echo (FFE) pulse sequence (EPI based, 3D) with retrospective electrocardiogram (ECG)-triggering. The acquisition voxel size was kept as close as possible to 3 × 3 × 3 mm3. FOV and number of slices (i.e., the 3D volume) was adapted to the subject’s size. Scan parameters: TE 3.5 ms, TR 10 ms, flip angle 10°, FOV 400 mm, number of signal averages 1. VENC 150 cm/sec. Acceleration was achieved by EPI factor 5. Free breathing was allowed, and no respiratory motion compensation was used. Number of slices was 40, temporal resolution used was 40 ms and the number of reconstructed phases was set to 30. EPI sequence is detailed in the Additional file 1: Table S1 as previously validated [21].
5. Modified Look-Locker inversion recovery (MOLLI) to determine the T1-inversion time [only done in the patient population]
6. LGE imaging was done at 15-min from gadolinium-based contrast injection in MI patients only. LGE-imaging was done by phase sensitive inversion recovery (PSIR) spoiled gradient echo (GE) sequence as per previous published methods [22, 23]. PSIR sequence details are as follows: SENSE factor 1.7, typical TE/TR of 3.0/6.1 msec, flip angle of 25°, slice thickness of 10 mm and with Look-Locker scout determined T1-inversion time. [only done in the patient population]

### 4D flow data reconstruction method

4D flow encoding was performed by standard 4-point encoding. Online/offline 4D flow data quality assurance checks were done as per previous published literature [21].

### 4D flow error corrections and quality checks

The effects of concomitant gradient terms were compensated using Maxwell correction methods by the CMR scanner. Remaining background errors were corrected by the local phase correction (LPC) filter on the CMR scanner performed in two-dimensional way- slice by slice. The LPC is a magnitude-weighted spatial low pass filter; pixels that are expected to be part of the static background are used with a higher weight than noisy background pixels or pixels that are expected to contain flow to determine the local phase offset. LPC uses surrounding tissue to determine “static” areas [24, 25].

All 3D phase contrast data sets were investigated for phase aliasing artefacts. If present then phase unwrapping was performed as per previously published guidelines on phase-contrast methods [26]. Additionally, any spatial misalignment of 4D flow data to cine imaging was corrected before any flow analysis was performed. This was done by visualizing streamlines in 4-chamber view at peak systole and repositioning them over descending aorta. Similar checks were done during diastole in 4-chamber and 2-chamber views for peak mitral inflow streamlines.

### Image analysis

All images were analysed by PG (3-years-experience in advanced CMR techniques, PG did the blinded tissue characterisation for infarct location and size for all MI cases), SC (1-year-experience in advanced CMR techniques, SC did the blinded volumetric assessment) and RVDG (> 5 years experience in advanced CMR techniques, RVDG did the blinded LV flow KE mapping). Images were evaluated offline using research software (MASS; Version 2016EXP, Leiden University Medical Center, Leiden, The Netherlands). LV volumes, stroke volume, cardiac index, EF and infarct location on LGE imaging were determined according to standard methods. Acute and chronic infarct size were determined by the full-width at half-maximum semi-automated technique [27].

The degree of LV systolic impairment was categorised as follows: normal EF in controls, normal LVEF. In patients with MI, preserved LVEF was ≥55%, mildly impaired LVEF 45–54%, moderately impaired LVEF 35–44% and severely impaired LVEF < 35%" In patients with MI.

### Kinetic energy mapping

For calculation of LV blood flow KE parameters, the LV volumetric mesh was resliced into short-axis sections of 2 mm thickness and pixel spacing equal to the original reconstructed pixel size of the short-axis cine acquisition (1.0–1.2 mm). This high-resolution LV mesh is constructed by representing the mesh in cylinder coordinates. The LV radius for a given angle and LV level is derived by linear interpolation. Correction for translational and rotational misalignment between the short-axis cine and the 4D Flow CMR acquisition was performed using automated image registration as previously described [28].

For each volumetric element (voxel) the KE was computed as $\mathrm{KE}=\frac{1}{2}\ {\rho}_{blood}.{V}_{voxel}.{v}^2$, with *ρ*_*blood*_ being the density of blood (1.06 g/cm^3^), *V*_*voxel*_ the voxel volume and *v* the velocity magnitude. For each phase, the total KE within the LV was obtained by summation of the KE of every voxel. Time-resolved kinetic energy curves were generated to derive physiologically relevant parameters.

LV blood flow KE parameters which were derived for the complete cardiac cycle are described in Table 1/Fig. 1.

**Table 1:** Description of all left ventricular (LV) kinetic energy (KE) flow parameters investigated in this study. In this study all primary KE parameters are indexed to LV end diastolic volume (EDV) and expressed as KEi_EDV_

Global KE parameters	
LV KE^a^	The average KE of the LV flow for the complete cardiac cycle.
In-plane KE (%)	The proportion of in-plane KE of the LV, from the complete LV flow, for the complete cardiac cycle.
Minimal KE^a^	The minimal KE of the LV flow at any time point during the complete cardiac cycle.
Systolic KE parameters	
Systolic KE^a^	The average KE of the LV flow during systole.
Diastolic KE parameters	
Diastolic KE^a^	The average KE of the LV flow during diastole.
Peak E-wave KE^a^	The peak KE of the LV flow during early diastolic filling.
Peak A-wave KE^a^	The peak KE of the LV flow during late diastolic filling.
Time difference, TD	This is the time difference of peak E-wave flow KE from base to mid-ventricle.

^a^These KE parameters were normalised to LVEDV and presented as KEi_EDV_

The novel KE parameters studied in this study are detailed below:

### In-plane KE

We define the in-plane KE as the sum of all kinetic energy in the x-y direction, in the short-axis LV from base to apex. In this study, the in-plane KE is represented as a percentage of the total LV KE, this negates the issue with volume normalisation. Most of the blood flow in the LV should be in the through-plane direction – both in systole (towards aortic valve) and during diastole (towards apex from the mitral valve) and an increase in the proportion of in-plane flow may be associated with pathological flow. Hence, in this study, the in-plane KE parameter was computed mainly to better understand the in-plane flow dynamics within the LV cavity.

### Time difference (TD)

We also computed the time difference (TD) to peak early mitral inflow velocity (E-wave) from the base of the LV to mid-ventricle. This transit time or TD should be higher if the mitral valve propagation velocity (Vp), as measured by M-Mode echocardiography is lower [29]. Hence, the transit time of the peak KE from base to mid-ventricle, described as the TD in this study, may represent delayed filling.

### Minimal KE

This parameter simply represents the minimal KE of the LV flow at any time point during the complete cardiac cycle. We computed this parameter mainly to understand how KE is preserved in health versus disease. The minimal KE of the LV is likely to be happen when the haemodynamic forces are minimal, and LV is not moving. A rise in the minimal KE may be influenced by heterogenous haemodynamic forces described in the LV fluid dynamics model secondary to dyssynchronous or late systolic contractility seen in infarcted segments [30].

To allow comparison between patients and healthy subjects, all KE parameters were normalized to the LV end-diastolic volume (EDV) and accordingly reported in μJ/ml. In sub-analysis, we also normalised the KE parameters to the stroke volume to develop insight into KE spent per unit of stroke volume.

### Intra-/inter-observer variability

For inter-observer variability, SC and RVDG contoured the short-axis LV cine volumetric stack in 10 random healthy control subjects and 10 random MI patients, blinded to each other’s analysis. Automated KE parameters were again generated using the endocardial contours from second observer. For intra-observer variability, SC re-analysed the LV short-axis cines for the same 10 healthy control subjects and 10 MI patients MI after 3 months. Akin to inter-observer variability, automated KE parameters were generated using the new endocardial contours by the same observer using the time-resolved methods previously described [31].

### Statistical analysis

Statistical analysis was performed using SPSS® Statistics 21.0 (International Business Machines, Armonk, New York, USA). Continuous measurements are presented as mean ± standard deviation. Quantitative flow imaging parameters expected to be non-parametric were presented as median and inter-quartile ranges (IQR). Demographic comparisons were performed with an independent samples t-test. Comparisons between two groups with non-parametric data were made using Mann–Whitney U test. Intra−/inter-observer reliability tests were done by inter-class correlation coefficient. In different categories of LVEF, post hoc analysis was done by Kruskal-Wallis H test. Association of infarct size to KE parameters was done by Spearman’s rank correlation coefficient test. In multi-variate analysis, a forward-conditional method was used for regression and parameters with statistical significance from one-way analysis (*p* < 0.05) were chosen for multi-variate analysis. A *p*-value < 0.05 was considered statistically significant.

## Results

### Demographic characteristics

Of the 55 patients with acute and chronic MI patients initially identified and recruited, 48 patients completed the full study protocol (Fig. 2). Controls and patients were matched for age, (52 ± 11 years versus 57 ± 11 years, *P* = 0.1), gender and body surface area (Table 2).

**Table 2:** Demographics and CMR parameters in healthy controls versus MI patients

	Controls (*n* = 20)	MI Patients(*n* = 48)		*P*-value†	*P*-value‡
		pEF (12)	rEF (36)		
Age (years)	52.3 ± 11.2	57.1 ± 10	57.3 ± 12	0.10	0.93
Gender (Male, n)	12	9	30	0.07	0.53
Body surface area	1.8 ± 0.2	1.9 ± 0.3	1.98 ± 0.4	0.69	0.68
Acute MI (n)	–	8	27	–	0.58
Heart rate (bpm)	65 ± 12	58 ± 10	66 ± 14	0.75	0.045
Standard CMR clinical parameters					
LVMi (g/m^2^)	51.0 ± 10.2	57.7 ± 16.6	62 ± 19	0.02	0.46
LVEDVi (ml/m^2^)	81.6 ± 15.6	80 ± 10	103 ± 30	0.02	0.01
LVESVi (ml/m^2^)	30.0 ± 7.4	32 ± 6	64 ± 30	< 0.01	< 0.01
SVi (ml/m^2^)	51.6 ± 10.7	48 ± 5	38 ± 9	< 0.01	< 0.01
EF (%)	63.1 ± 5.6	60 ± 3	39 ± 11	< 0.01	< 0.01
IS (% of LV)	–	12.3 ± 9	25 ± 12	–	< 0.01
CI (L/min/m2)	3.3 ± 0.9	2.7 ± 0.47	2.5 ± 4.6	0.03	0.11
LV KEi_EDV_ parameters					
LV (μJ/ml)	8.5 ± 2.7	7 ± 4	6.3 ± 3	0.02^a^	0.11
Minimal (μJ/ml)	0.95 ± 0.63	0.5 ± 0.3	0.8 ± 0.6	0.02^a^	0.02
Systolic (μJ/ml)	9.2 ± 3.8	8.4 ± 6	6.4 ± 2	< 0.01^a^	< 0.01
Diastolic (μJ/ml)	7.5 ± 4.2	7 ± 3	6.3 ± 3.5	0.29	0.92
E-wave (μJ/ml)	22.0 ± 10.2	19 ± 8	15 ± 9	0.02^a^	0.30
A-wave (μJ/ml)	12.5 ± 5.6	14 ± 9	10.6 ± 7	0.22	< 0.01
In-plane LV (%)	36.5 ± 7.2	31 ± 4.4	38 ± 8	0.84	< 0.01
TD (msecs)	11 ± 31	33.6 ± 12	34.2 ± 52	< 0.01^a^	0.21

Data is presented as mean ± standard deviation (SD) or count (n) for demographics and standard CMR parameters and median ± inter-quartile range (IQR) for KE parameters. LV measurements are indexed to body surface area

All KE values are indexed for LV end-diastolic volume

*LVEDVi* Left ventricular end diastolic volume (indexed), *LVESVi* Left ventricular end systolic volume (indexed), *LVMi* left ventricular mass (indexed), *MV* mitral valve, *SVi* stroke volume (indexed)

^a^parameters which went in forward regression

†*P*-values comparing controls versus MI patients

‡= *P*-values comparing pEF group versus rEF group

### Baseline CMR data

Heart rate was comparable between MI patients and healthy controls (65 ± 12 bpm versus 64 ± 13 bpm, *P* = 0.75). Most baseline volumetric CMR parameters were significantly higher in MI patients than healthy controls (Table 2). Infarct size was similar among patients with acute and chronic MI (24.3 ± 14% vs. 19.6 ± 11%, *P* = 0.21). The majority of patients had anterior MI (*n* = 35) versus inferior/posterior MI (*n* = 13). The 4D flow CMR acquisition time was 8 ± 2 min.

### Global LV flow KE in MI

**Fig. 3:**
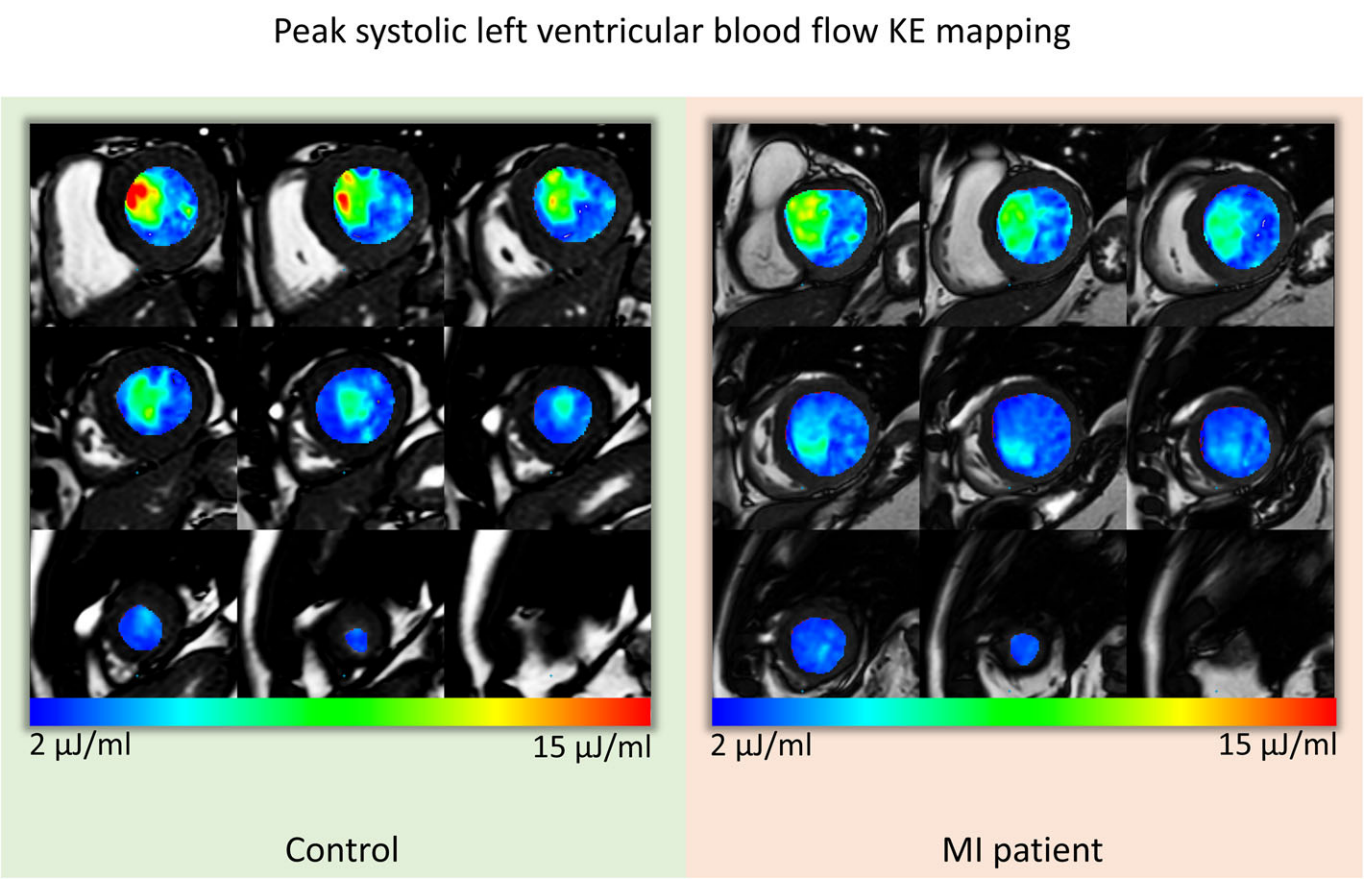
Left ventricular (LV) blood flow KE mapping. The short-axis whole LV blood flow KE maps represented here are during peak systole. The KE of blood flow in the out-flow tract is higher in the control versus the myocardial infarction (MI) patient

**Fig. 4:**
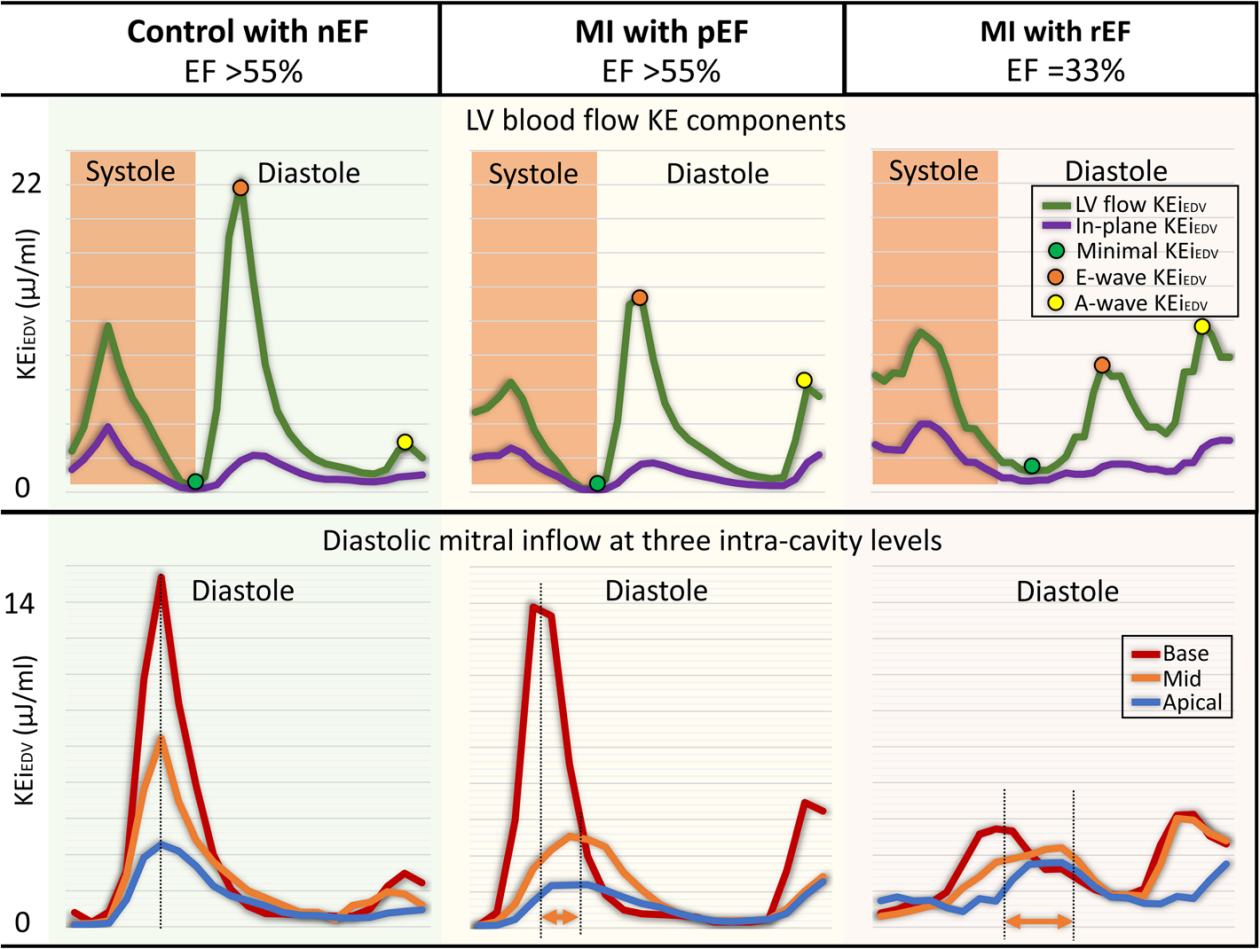
Case examples of KE curves in control and two MI patients with preserved LVEF and reduced LVEF. First panel: The total KE of the LV is reduced in MI patients. The minimal KE demonstrates an increase in MI patient with reduced LVEF. Second panel: Diastolic KE curves at different levels of the LV (base, mid-ventricular and apex). The diastolic KE curves demonstrate blunting of peak E-wave KE in the two patient groups. Time differences to peak E-wave KE propagation increase in each group (orange arrows between the two dotted black lines). EF = ejection fraction, nEF = normal ejection fraction, pEF = preserved ejection fraction, KE = kinetic energy, LV = left ventricle and MI = myocardial infarction

Average LV flow KEi_EDV_ was significantly higher in healthy controls than in MI patients (8.5 ± 3 μJ/ml versus 6.5 ± 3 μJ/ml, *P* = 0.02) (Table 2) (Fig. 3). Similarly, LV flow minimal KEi_EDV_ was significantly lower in patients. The proportion of in-plane KE was not significantly different in healthy controls and MI patients. Diastolic peak E-wave KEi_EDV_ was significantly lower in MI patients compared to the healthy controls (*P* < 0.05). Time differences to peak E-wave KEi_EDV_ were significantly higher in MI patients compared to healthy controls (11 ± 31msecs versus 34 ± 29msecs, *P* < 0.01) (Fig. 4).

In logistic regression analysis of all the significantly different KEi_EDV_ parameters in MI patients (Table 2), LV blood flow systolic KEi_EDV_ demonstrated the strongest association with the presence of MI (Beta = − 0.24, standard error = 0.08, *P* < 0.01).

### Association with cardiac haemodynamic parameters

Kinetic energy parameters that were significantly associated with heart rate included the following: LV, minimal and diastolic KEi_EDV_ (*P* < 0.05) (Table 3). For these parameters, between EF group analysis was adjusted to the heart rate.

**Table 3:** Association of LV kinetic energy parameters to LV haemodynamic parameters

KE parameters	Heart rate		Stroke volume		Cardiac index	
	rho	*P*-Value	rho	*P*-Value	rho	*P*-Value
LV KEi_EDV_ (μJ/ml)	0.31	**0.01**	0.20	0.11	0.42	**< 0.01**
Minimal KEi_EDV_ (μJ/ml)	0.43	**< 0.01**	− 0.07	0.58	0.06	0.64
Systolic KEi_EDV_ (μJ/ml)	0.05	0.70	0.45	**< 0.01**	0.48	**< 0.01**
Diastolic KEi_EDV_ (μJ/ml)	0.35	**< 0.01**	0.03	0.83	0.28	**0.02**
E-wave KEi_EDV_ (μJ/ml)	0.02	0.90	0.31	**0.01**	0.30	**0.01**
A-wave KEi_EDV_ (μJ/ml)	0.14	0.24	−0.01	0.92	0.24	0.05
In-plane LV (%)	0.06	0.63	− 0.28	**0.02**	−0.29	**0.02**
TD (msecs)	0.09	0.45	−0.31	**0.01**	−0.23	0.06

Bold text = significant *P*-values

*KE* kinetic energy, *LV* left ventricle, *Rho* Spearman rank correlation coefficient

Kinetic energy parameters that were significantly associated with stroke volume included the following: systolic and E-wave KEi_EDV_, the proportion of in-plane KE and TD (*P* < 0.05) (Table 3). In sub-group analysis, we did not identify significant association between stroke volume and systolic KEi_EDV_ in the controls (*R* = 0.31, *P* = 0.17). This association was mainly driven by variation of stroke volume in the MI cohort (*R* = 0.39, *P* < 0.01). However, KE parameters associated with cardiac index included the following: LV, systolic, diastolic, E-wave KEi_EDV_ and in-plane KE (*P* < 0.05).

### LV systolic impairment and flow KE

**Fig. 5:**
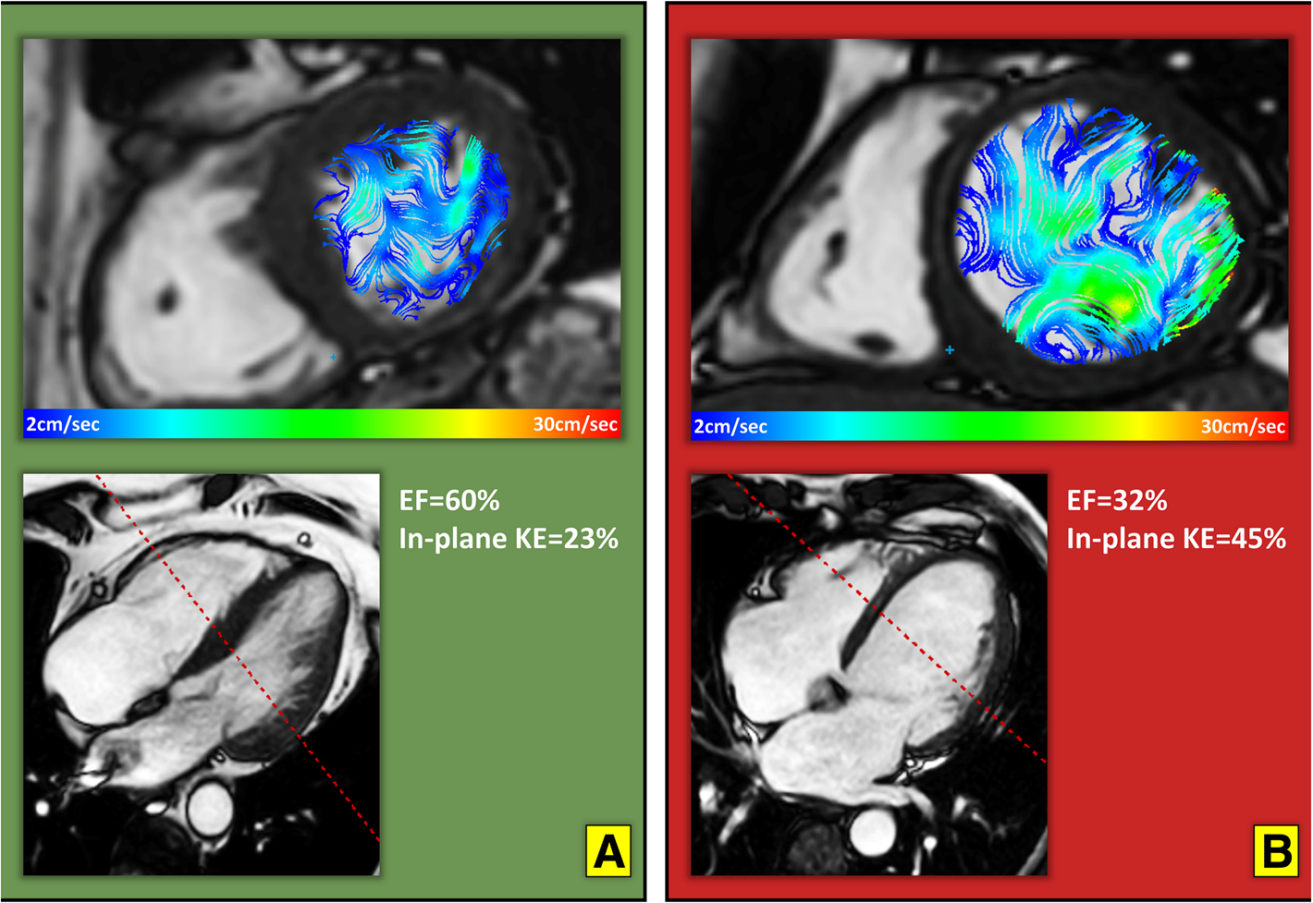
In-plane velocity streamlines in two case examples from the study demonstrating higher in-plane (xy-axis) flow during diastasis in a patient with myocardial infarction and worse EF (Panel **b**) versus an MI patient with a smaller infarct and preserved LVEF (Panel **a**). The in-plane KE was computed as the sum of all KE in the x-y direction, in the short-axis left ventricle from base to apex. It is represented as a percentage of the total LV KE. KE = kinetic energy and EF = ejection fraction

**Fig. 6:**
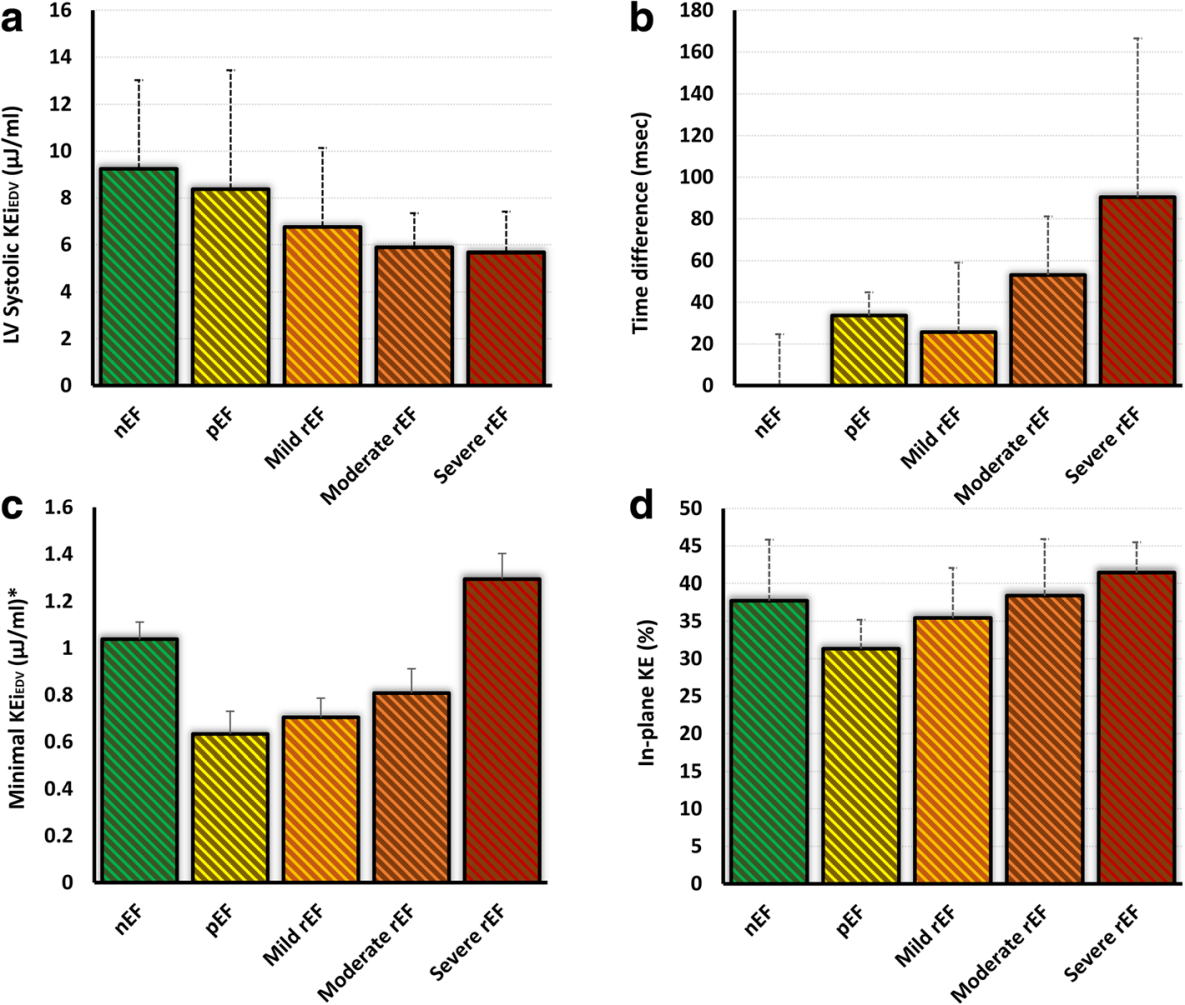
Bar charts of left-ventricular (LV) flow kinetic energy parameters and their variations within ejection fraction (EF) sub-groups. The LV systolic KEi_EDV_ (Panel **a**) demonstrated reduction with worsening LVEF versus TD (Panel **b**) which demonstrated significant increase with worsening LVEF. Note the U-shaped pattern of minimal (Panel **c**) and in-plane (Panel **d**) KE of the LV. (bars = median; error bars = inter-quartile range). KE = kinetic energy, LV = left ventricle, nEF = normal ejection fraction, pEF = preserved ejection fraction, Mild rEF = mildly reduced ejection fraction, Moderate rEF = moderately reduced EF and Severe rEF = severely reduced EF

Comparison of LV flow KE parameters (raw, normalised to end-diastolic volume and normalised to stroke volume) with the degree of LV systolic impairment are detailed in Table 4. Several LV flow KEi_EDV_ parameters demonstrated significant differences between groups of patients, defined by the degree of LV systolic impairment (*P*-value< 0.05). These included: minimal, systolic, in-plane KEi_EDV_ parameters and TD to peak E-wave KE propagation. The proportion of in-plane KE and minimal KEi_EDV_ parameters were significantly reduced inpreservedpEF patients when compared to normal EF healthy controls but were increased compared with controls in patients with worsening EF (Figs. 5, 6c and d). In exploratory analysis, there was a modest association between the proportion of in-plane KE and minimal KEi_EDV_ parameters (*R* = 0.35, *P* < 0.01). The systolic KEi_EDV_ decreased significantly with worsening EF (Fig. 6a). TD to peak E-wave KE propagation demonstrated a linear increasing trend with worsening EF (*P* < 0.01).

**Table 4:** Post-hoc analysis of several LV KE flow parameters in different LV function sub-groups

	(a) normal EF (*n* = 20)	(b) preserved EF (*n* = 12)	(c) Mild (*n* = 16)	(d) Moderate (*n* = 10)	(e) Severe (*n* = 10)	*P*-value
Kinetic energy parameters normalised to LVEDV (KEi_EDV_)						
LV^#^	8.5 ± 2.7	7 ± 2.9	6.3 ± 2.9	6.1 ± 1.2	6.8 ± 3.5	0.10
Minimal^#^	0.95 ± 0.63^b, c^	0.53 ± 0.29^a, e^	0.56 ± 0.32^a, e^	0.76 ± 0.37^e^	1.48 ± 0.54^b, c, d^	< 0.01
Systolic	9.2 ± 3.8^c, d, e^	8.4 ± 5.1^d, e^	6.8 ± 3.2^a, d^	5.9 ± 1.5^a, b, c^	5.7 ± 1.7^a, b^	< 0.01
Diastolic^#^	7.5 ± 4.2	6.9 ± 2.4	5.6 ± 2.5	6.3 ± 2.3	7.5 ± 3.4	0.30
Peak E-wave	22 ± 10.2	19.1 ± 6.9	14.1 ± 9.2	14.6 ± 7.3	17.3 ± 9.4	0.13
Peak A-wave	12.5 ± 5.6^e^	13.7 ± 7.7^e^	11.4 ± 5.5	10.9 ± 6.2	8 ± 6.2^a, b^	0.10
In-plane (%)	36.5 ± 7.2^b, e^	31.3 ± 3.9^a, d, e^	35.5 ± 5.6^e^	38.4 ± 7.5^b^	41.4 ± 4^a, b, c^	< 0.01
TD (msec)	11 ± 31^b, d, e^	34 ± 11^a, e^	26 ± 33^d, e^	53 ± 28^a, c^	90 ± 76^a, b, c^	< 0.01
Kinetic energy parameters normalised to SV (KEi_SV_)						
LV^#^	13 ± 5^e^	12 ± 7^e^	12 ± 5^e^	18 ± 6^e^	29 ± 19^a, b, c, d^	< 0.01
Minimal^#^	1.4 ± 0.9^e^	0.9 ± 7^e^	1.1 ± 0.9^e^	1.9 ± 1.5^e^	6 ± 4^a, b, c, d^	< 0.01
Systolic	15 ± 5^e^	15 ± 10^e^	14 ± 6^e^	16 ± 6^e^	22 ± 5^a, b, c, d^	0.03
Diastolic^#^	11.6 ± 6^d, e^	11.6 ± 5^e^	11.6 ± 5^e^	17 ± 10^a, e^	34 ± 15^a, b, c, d^	< 0.01
Peak E-wave	35 ± 14^e^	33 ± 15^e^	29.5 ± 19^e^	39 ± 27	62 ± 52^a, b, c^	0.02
Peak A-wave	19 ± 10^e^	23.5 ± 13	22 ± 1^e^	32 ± 17	33 ± 27^a, c^	0.03
Kinetic energy parameters without normalisation						
LV^#^	1.3 ± 0.6^e^	1.3 ± 1^e^	1.1 ± 0.7^e^	1.3 ± 0.5^e^	1.9 ± 0.7^a, b, c, d^	0.15
Minimal^#^	0.14 ± 0.07^b, e^	0.08 ± 0.09^a, d, e^	0.13 ± 0.1^e^	0.15 ± 0.1^b, e^	0.36 ± 0.08^a, b, c, d^	< 0.01
Systolic	1.45 ± 0.8	1.5 ± 1.3	1.3 ± 0.9	1.2 ± 0.3	1.7 ± 0.65	0.27
Diastolic^#^	1.14 ± 0.8^e^	1 ± 0.6^e^	1 ± 0.6^e^	1.3 ± 0.7	2.2 ± 0.9^a, b, c^	< 0.01
Peak E-wave	3.5 ± 1.5	3 ± 1.5	2.3 ± 1.6	3 ± 2.2	4.3 ± 2.7	0.16
Peak A-wave	1.7 ± 0.6	2 ± 1.2	1.9 ± 1.1	2.1 ± 1.4	2.4 ± 2.1	0.35

All data is represented as median ± inter-quartile range. Significant differences within groups represented as superscript

*nEF* normal ejection fraction in controls with no MI, *pEF* MI patients with preserved ejection fraction, *LV* left ventricle, *TD* time difference of peak E-wave KE propagation from base to mid-ventricle

^#^*P*-value is adjusted for the co-variate: heart rate

### Infarct size and flow KE

**Fig. 7:**
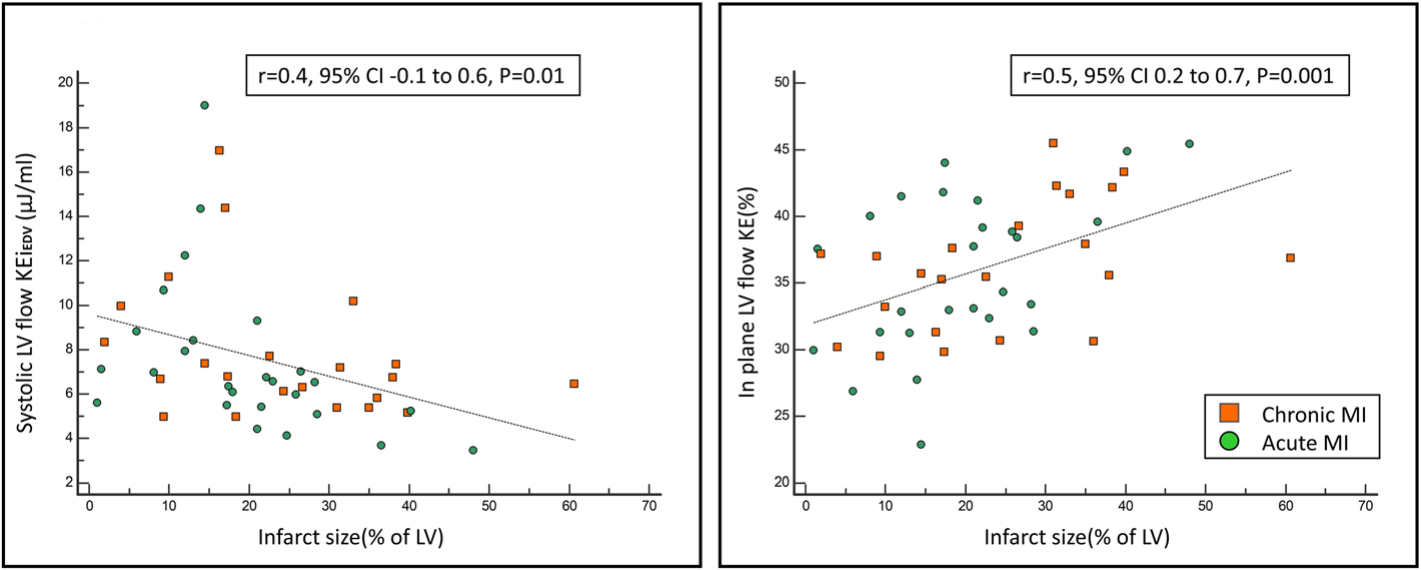
Scatter plots of infarct size and its positive association to in-plane KE and negative association to LV systolic flow KE. KE = kinetic energy, LV = left ventricle and MI = myocardial infarction

Infarct size was significantly associated with minimal, systolic and peak A-wave KEi_EDV_, in-plane KE and the TD for peak E-wave KE (Table 5). Infarct size was negatively associated with systolic KEi_EDV_ (rho = − 48, *P* = 0.01) and positively associated with the proportion of in-plane KE (rho = 0.5, *P* = 0.001) (Fig. 7). TD of peak E-wave KE propagation had a modest positive association with MI size (rho = 0.33, *P* = 0.02). In multiple regression analysis, in-plane KE was independently associated with infarct size (Beta = 1.1, *P* = 0.001) (Table 5).

**Table 5:** LV flow KEi_EDV_ parameters and their association to the infarct size in patients with MI

	Univariate					Multivariate			
	rho	Beta	95% CI	SE	*P*-value	Beta	95% CI	SE	*P*-value
LV KEi_EDV_	−0.29	−1.4	−3 to 2	0.8	0.09				
Minimal KEi_EDV_	0.26	0.01	0 to 0.2	0.005	**0.04**				
Systolic KEi_EDV_	−0.48	− 0.1	− 0.2 to − 0.02	0.03	**0.01**				
Diastolic KEi_EDV_	−0.07	− 0.02	− 0.07 to 0.04	0.03	0.59				
Peak E-wave KEi_EDV_	−0.24	−0.14	− 0.3 to 0.03	0.08	0.09				
Peak A-wave KEi_EDV_	−0.37	−0.14	− 0.2 to − 0.02	0.05	**0.02**				
In-plane LV (%)	0.5	1.1	0.5 to 1.7	0.3	**0.001**	1.1	0.5 to 1.7	0.3	0.001
TD (msec)	0.33	1.1	0.2 to 2	0.4	**0.02**				

Bold *P*-values were selected for multivariate analysis

*CI* confidence interval, *LV* left ventricle, *SE* standard error, *rho* Spearman’s rank correlation coefficient

### Anterior versus inferior/posterior infarction

Thirty-five patients (73%) had anterior MI and 13 (27%) had inferior/posterior MI. CMR baseline parameters were similar in both infarct locations (Table 6). Infarct size was significantly larger in patients with anterior MI (14.4 ± 12% vs 23 ± 18%; *P* < 0.01). Controlling for infarct size, no significant differences were noted for the baseline CMR parameters and the different parameters of KEi_EDV_ between anterior and inferior/posterior MI.

**Table 6:** Kinetic energy distribution in patients with anterior and inferior infarcts. Units for all KE parameters are μJ/ml

	Inferior MI (*n* = 13)	Anterior MI (*n* = 35)	*P*-value
Baseline CMR Parameters			
LVEDVi (ml/m^2^)	83 ± 19	92 ± 26	0.56✝
LVESVi (ml/m^2^)	45 ± 24	51 ± 24	0.8✝
SVi (ml/m^2^)	41 ± 8	41 ± 15	0.26✝
EF (%)	49 ± 10	43 ± 21	0.87✝
EDMi (g/m^2^)	54 ± 8	56 ± 24	0.41✝
IS (%)	14.4 ± 12	23 ± 18	< 0.01
LV KE parameters			
LV KEi_EDV_	6.6 ± 3.7	6.4 ± 2.2	0. 78✝
Minimal KEi_EDV_	0.55 ± 0.63	0.74 ± 0.48	0.17✝
Systolic KEi_EDV_	7.9 ± 3.5	6.4 ± 2.0	0.33✝
Diastolic KEi_EDV_	6.7 ± 3.0	6.5 ± 3.0	0.68✝
Peak E-wave KEi_EDV_	15.6 ± 10.7	16.6 ± 9.9	0.9✝
Peak A-wave KEi_EDV_	11.6 ± 3.9	10.7 ± 7.3	0.63✝
In-plane LV (%)	33 ± 6	38 ± 8	0.62✝
TD (msecs)	30 ± 40	35 ± 38	0.28✝

All data is represented as median ± inter-quartile range

*EF* left ventricular ejection fraction, *IS* infarct size, *LV* left ventricle, *LVEDVi* Left ventricular end diastolic volume (indexed), *LVESVi* Left ventricular end systolic volume (indexed), *LVMi* left ventricular mass (indexed), *MR* mitral regurgitation, *SVi* stroke volume (indexed), *TD* time difference of peak E-wave KE propagation from base to mid-ventricle

✝ *P*-value controlled for infarct size

### Acute versus chronic MI

Baseline demographics were comparable between patients with either acute or chronic MI (Table 7). From the baseline CMR parameters, only stroke volume was significantly higher in chronic MI versus acute (37 ± 10 ml/m2 versus 44 ± 8 ml/m2, *P*-value = 0.02). KEi_EDV_ parameters and LV EF were comparable in both groups.

**Table 7:** Comparison of study results in acute versus chronic myocardial infarction

	Acute MI (*n* = 22)		Chronic MI (*n* = 26)		*P*-value
	Mean/Median	SD/IQR	Mean/Median	SD/IQR	
Age (years)	55.00	11.36	59.27	10.98	0.19
Gender (Male)					0.53
Body surface area	2.00	0.28	1.95	0.49	0.69
Heart rate (bpm)	68	16	61	10	0.10
Baseline CMR results					
IS (% of LV)	24	14	20	11	0.22
LVEDVi (ml/m^2^)	94	29	99	26	0.54
LVESVi (ml/m^2^)	57	28	55	30	0.86
SVi (ml/m^2^)	37	10	44	8	**0.02**
LVMi (g/m^2^)	63	22	59	14	0.53
EF (%)	42	12	46	14	0.24
Kinetic Energy Mapping results					
LV KEi_EDV_	6.7	2.5	6.4	2.0	0.71
Minimal KEi_EDV_	0.8	0.7	0.6	0.5	0.53
Systolic KEi_EDV_	6.7	2.3	6.7	2.9	0.68
Diastolic KEi_EDV_	6.9	3.5	6.1	2.7	0.79
Peak E-wave KEi_EDV_	15.6	11.7	16.6	8.7	0.76
Peak A-wave KEi_EDV_	11.9	3.9	10.7	8.0	0.51
In-plane LV (%)	37.2	7.2	33.1	8.3	0.75
TD (msecs)	34.0	27.9	33.3	25.6	0.82

Data is presented as mean ± standard deviation (SD) or count (n) for demographics and standard CMR parameters and median ± inter-quartile range (IQR) for KE parameters. LV measurements are indexed to body surface area

All KE values are indexed for LV end-diastolic volume. Units for all KE parameters are μJ/ml

*LVEDVi* Left ventricular end diastolic volume (indexed), *LVESVi* Left ventricular end systolic volume (indexed), *LVMi* left ventricular mass (indexed), *MV* mitral valve, *SVi* stroke volume (indexed)

### Kinetic energy of blood flow per stroke volume

**Fig. 8:**
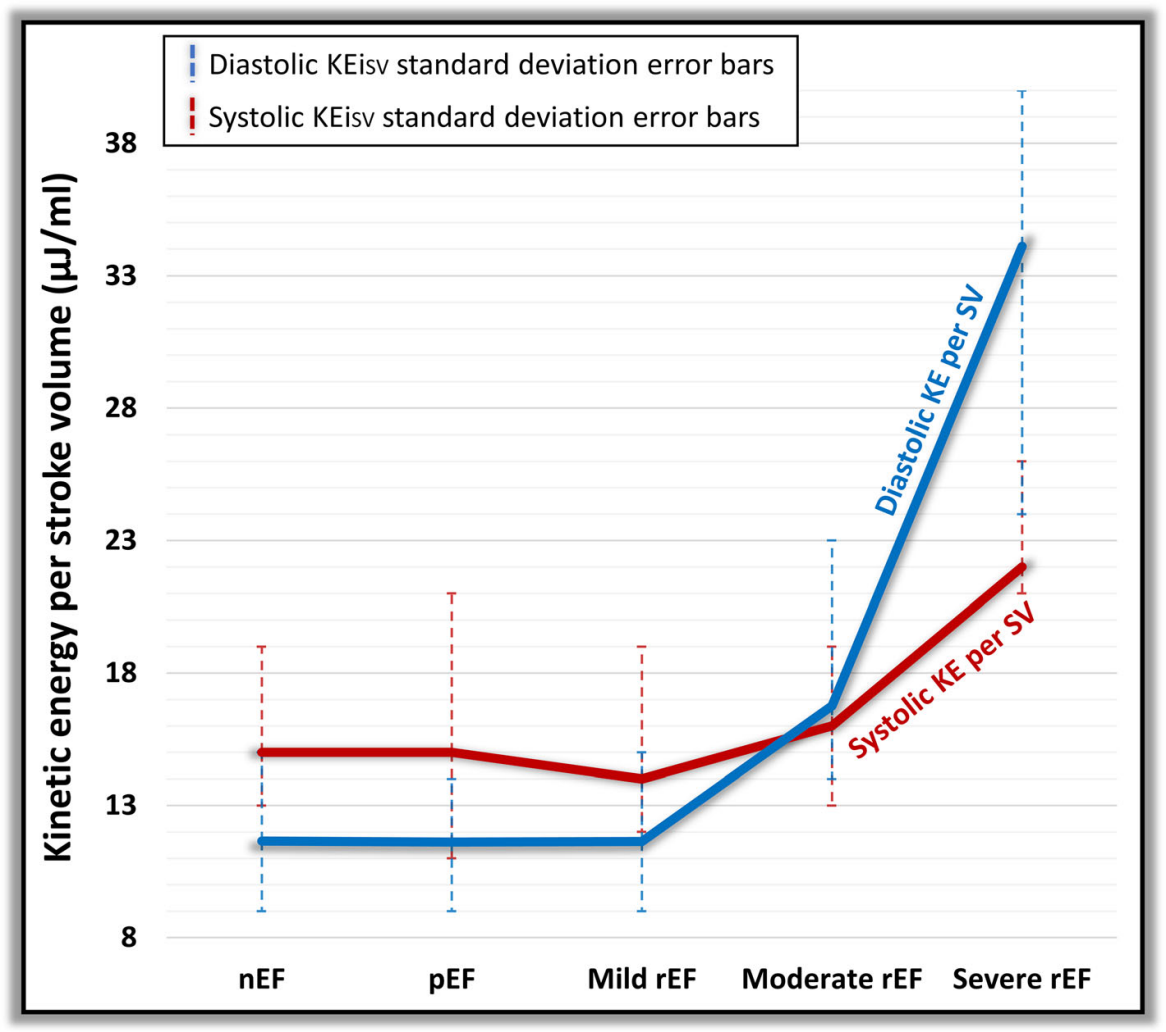
Comparison of systolic and diastolic kinetic energy per LV stroke volume in different categories of LV impairment. KE = kinetic energy, nEF = normal ejection fraction, pEF = preserved ejection fraction, Mild rEF = mildly reduced ejection fraction, Moderate rEF = moderately reduced EF, Severe rEF = severely reduced EF and SV = stroke volume

All KEi_SV_ parameters (LV, minimal, systolic, diastolic, E-wave and A-wave) demonstrated significant rise between different groups of EF (*P* < 0.05) (Table 4). We noted that rise in KE in diastole per stroke volume over took systolic KE per stroke volume from the moderately impaired LV group. Hence, in moderately and severely impaired LV, there is more KE per stroke volume in diastole than systole (Fig. 8).

### Intra-observer and inter-observer variability

Intra-observer and inter-observer variability results for global KE parameters are detailed in the online Additional file 1: Table S2. In summary, all global KE parameters demonstrated excellent intra-class correlation coefficient (ICC) (average 0.99, *P*-value> 0.9). In addition, the mean bias (%) for all KEi_EDV_ parameters was 2 ± 9% for intra-observer tests and 3 ± 9% for inter-observer tests. TD to peak E-wave flow KE also demonstrated very high intra-class correlation coefficient (ICC = 0.94, 95% CI 0.88 to 0.97).

## Discussion

In this study, we mapped different parameters of LV blood flow KE in MI patients and matched healthy control subjects. We found that the majority of LV blood flow KEi_EDV_ parameters are reduced in patients with MI. In addition, decrease in systolic KEi_EDV_ of the LV blood flow is independently associated with the presence of MI and the proportion of in-plane KE of the LV blood flow is independently associated with the infarct size. Another important finding of this study was that worsening degree of LV systolic function is associated TD of peak E-wave KE propagation. MI patients with preserved EF have significantly reduced minimal KEi_EDV_, the proportion of in-plane KE and increased TD of peak E-wave KE propagation when compared with healthy controls with nEF. Kinetic energy per stroke volume revealed higher systolic KE versus diastolic KE in normal EF, preserved EF and mildly reduced EF groups. However, patients with moderate and severely reduced EF demonstrated reversal and had higher diastolic KE per stroke volume than systolic KE. Finally, global LV blood flow KE parameters demonstrated good intra−/inter-observer variability.

### Global LV flow KE in MI

In the present study, the global LV flow KE_iEDV_, which in essence is the global density of the velocity profile of intra-cavity blood flow, was significantly lower in patients with MI than controls. This is in keeping with results by Kanski et al., who found reduced indexed LV KE in patients with moderate-to-severe heart failure versus controls. (6.3 ± 2.2 μJ/ml vs 8.0 ± 2.1 μJ/ml, *p* = 0.025) [16]. Even though the diastolic parameter of LV KE was not significantly different in MI patients, the peak E-wave KE was significantly lower in MI patients. This would be consistent with early pre-clinical studies that have demonstrated that any condition that interferes with normal regional systolic function is expected to modify the pattern of the normal early diastolic intraventricular pressure gradients. The present study also showed that the presence of MI is most strongly associated with systolic KE_iEDV_ of LV blood flow. As a consequence of MI and regional hypo-contractility, there is reduced regional systolic mechanical force imparted on the intra-cavity blood consequently due to lowered pressure gradient between the LV and the aorta, the overall thrust of systolic blood flow is reduced as demonstrated by drop in the systolic blood flow energetics. This is also supported by the finding that LV stroke volume was only associated to systolic KEi_EDV_ in MI cohort and not in the controls.

### Delayed filling of the LV

During diastolic filling of the LV in health, we already know through M-mode Doppler echocardiography that blood flow into the LV cavity from base to apex happens very fast with a mitral valve flow propagation velocity (Vp) of above 50 cm/sec [32]. Even though in this study we did not measure the Vp, we measured the transit time (or TD in this study) for the peak E-wave to travel from base to mid-ventricle and hence, it is plausible to conclude that if the Vp will decrease, the time delay for the peak inflow will increase. Accordingly, we observed an increase in the time differences for peak E-wave KE propagation in MI patients compared with controls. This delay reflects restrictive LV filling and possible normalisation of intra-ventricular pressure gradients. Intra-ventricular pressure gradients represent a suction force and have been attributed to energy consuming, active LV relaxation [33, 34]. The significant increase in TDs in patients with MI and preserved EF versus controls with normal EF demonstrates that restrictive filling precedes any detectable systolic impairment. The TDs measured in this study offer physiological insight similar to mitral valve propagation velocity on echocardiography. Compared with mitral valve propagation velocity, the TDs measured here are semi-automatically computed and have low intra−/inter-observer variability. From the clinical perspective, this parameter may offer semi-automated and possible reliable assessment of the degree of LV diastolic impairment and the LV filling pressures. The reliability and the prognostic implications of using TD as a bio-imaging marker needs to be further tested in future studies.

### Association of LV flow KE to the degree of systolic impairment

The patterns of changes between these LV flow KE parameters to the degree of LV systolic impairment were different. The systolic KEi_EDV_ had a more linear trend to decrease with worsening EF. Decreased LV contractile function leads to impaired emptying and increased preload in subsequent cycles. Even if this causes stroke volumes to return to normal due to the Frank-Starling mechanism, the increased ventricular dimensions will cause the KEi_EDV_ to decrease.

The TDs for peak E-wave KE propagation demonstrated a more linear trend to increase with worsening EF. The minimal KEi_EDV_ and the proportion of in-plane KE significantly reduced from normal EF in healthy controls to MI patients with preserved EF and then had a strong trend to increase with decreasing EF – a finding that at first seems paradoxical.

We speculate that the in-plane KE of LV flow is made up of several in-plane blood flow movements during the complete cardiac cycle. These include: the physiological systolic in-plane movement of blood towards the outflow track during LV contraction, in-plane flow within the vortex in early and late diastolic filling. The systolic and vortex associated in-plane flow are plausibly decreased in MI patients with preserved EF as they have subtle mechanical dysfunction with preserved LV volumes. This finding would be consistent with other studies demonstrating alterations in diastolic inflow vortex strength in heart failure patients with preserved EF (HFpEF) [35]. However, progressive LV impairment and dilatation also causes increased sphericity, which will in turn change flow conditions inside the cavity to a ‘meta-stable’ state with a large, swirling vortex which encompasses the majority of the LV [36]. This vortex flow includes transversal thrusts which will show up as in-plane KE and become increasingly prominent as the ventricle remodels further. In addition, given the fact our reference spatial plane is the atrioventricular valve, the angular differences between the inflow and outflow direction which follows from the non-parallel orientation of the left atrium and aorta may result in significant outflow in-plane component.

As the pattern of changes to the degree of LV impairment was similar for in-plane KE and minimal KEi_EDV_, we speculate that the rise in the proportion of in-plane KE with worsening EF is associated with an increase in minimal KEi_EDV_. This is possibly explained by the fact that progressive LV impairment leads to LV dilatation and reduced filling of the LV causing an overall increase in the in-plane flow, which results in higher minimal KEi_EDV_ for the LV. This observation is further supported by the analysis of the KE parameters per stroke volume which demonstrate the all blood flow KE parameters rise significantly for each stroke volume with the degree of LV impairment. In addition, once the degree of LV impairment reaches the moderate zone, the systolic KE per stroke volume becomes lesser than the diastolic KE per stroke volume. This perhaps reflect the loss of mechanical push by LV in systole combined with restrictive early filling in diastole associated with high velocities.

### Infarct characteristics and LV flow KE

Results from our study show that an increase in infarct size increases the in-plane KE of LV blood flow. Such pathological rise in the in-plane KE may exert heterogenous haemodynamic forces on the LV wall, possibly contributing to more dilatation and increase in endothelial dysfunction in the endocardium [37–40].

In our study, anterior infarcts were significantly larger than inferior infarcts. When adjusted for MI size, MI location (anterior versus inferior) was not significantly associated with LV function or blood flow KE parameters. In a previous study done by Kanski et al., in sub-group of patients with MI, multiple stepwise linear regression analysis, averaged LV KE was associated with LV end-diastolic volume and not IS. Similarly, in this study averaged LV KEi_EDV_ was not associated with IS (*P* = 0.09), however, in regression, the in-plane demonstrated independent association to the IS. This would indicate that some components of LV blood flow energetics are more influenced by infarct size than others and its associated mechanical dysfunction than its location.

### Future clinical applications

We have demonstrated that using 4D flow that it is possible to detect subtle changes in cardiac function after MI even in patients with preserved EF. Longitudinal prospective studies are needed to establish whether LV flow KE has incremental prognostic impact over established markers such as EF, scar burden and microvascular obstruction. Studies are also needed to establish whether changes in intra-cardiac flow can be altered by medical therapy. After MI, LV remodelling leads to progressive LV cavity dilatation and subsequent heart failure. Using 4D flow it will be possible to investigate these mechanisms and assess the impact of existing and potentially novel pharmacological interventions on these processes.

To compute LV blood flow KE parameters, time-resolved LV volumetric endocardial contours are used [31]. Hence, the main influence on LV energetics variation is mainly secondary to endocardial contour delineation. If this is done consistently and methodically for the complete cardiac cycle as in this study and previous published literature, it results in not only highly reproducible LV volumetric assessment but also LV blood flow energetics.

### Study limitations

Respiratory navigation was omitted for the 4D flow acquisition which could have influenced KE parameters. However, whole-heart 4D flow head-to-head comparison studies have also demonstrated that non-respiratory navigated acquisition of 4D flow is comparable to respiratory navigated acquisition for intra-cardiac KE quantification [41]. In addition, a recent study validated a non-respiratory navigated 4D Flow EPI acceleration sequence for clinical use [21]. The temporal resolution of the 4D flow was 40 ms, which may affect the quality of KE and TD assessment. MI patients were prescribed 4D flow post contrast versus healthy controls who didn’t receive any contrast. Post contrast 4D flow improves the signal-noise-ratio and may have introduced some bias when comparing controls versus patients. This study used the start of the cardiac cycle as defined by the scanner’s electrocardiogram to calculate TDs. Scanner’s electrocardiogram is not always correct, and this may result in less precise TD estimates. We used The LV geometry was defined by LV cine stack which was done using breath-hold technique while the 4D flow was done using free breathing. Hence, although spatial miss-registration was corrected for, other issues still remain including difference in heart rate and physiological conditions. This may have impact on the time-varying flow characteristics which could not be corrected for. Results from this study cannot be applied to patients with significant valvulopathy, cardiomyopathies and congenital heart disease.

## Conclusions

Post MI, reduction in LV function results in reduction in LV blood flow KE and is most strongly associated with the systolic LV blood flow KE. Increase in infarct size results in proportionate increase in the in-plane KE of LV blood flow. Degree of LV impairment is associated with TD of peak E-wave KE. Even in patient with preserved EF post MI, LV blood flow KE mapping demonstrated significant changes in the in-plane KE, the minimal KEi_EDV_ and the TD. Further longitudinal studies are warranted to investigate the long-term clinical significance of mapping LV blood flow KE parameters.

## Additional file

Additional file 1: Supplementary Material. (DOCX 30 kb)

## Abbreviations

2D: Two-dimensional
3D: Three-dimensional
4D: Four-dimensional
bSSFP: Balanced steady state free precession
CMR: Cardiovascular magnetic resonance
ECG: Electrocardiogram
EF: Ejection fraction
EPI: Echo-planar Imaging acceleration
FOV: Field of view
GE: Gradient echo
HFpEF: Heart failure with preserved ejection fraction
ICC: Intra-class correlation coefficient
IQR: Inter-quartile range
KE: Kinetic energy
LGE: Late gadolinium enhancement
LPC: Local phase correction
LV: Left ventricle/left ventricular
MI: Myocardial infarction
MOLLI: Modified Look Locker inversion recovery
PPCI: Primary percutaneous coronary intervention
PSIR: Phase sensitive inversion recovery
STEMI: ST-elevation myocardial infarction
TD: Time difference
TE: Echo time
TR: Repetition time
VP: Propagation velocity
