# Supplementary material for: Left ventricular blood flow kinetic energy after myocardial infarction - insights from 4D flow cardiovascular magnetic resonance
Source: J Cardiovasc Magn Reson. 2018 Aug 30;20:61. doi: 10.1186/s12968-018-0483-6 (PMC6117925; doi:10.1186/s12968-018-0483-6)
Supplement: Supplementary file 1 — Supplementary Material. (DOCX 30 kb) [file 12968_2018_483_MOESM1_ESM.docx]

**Supplementary Material**

**Contents**

1. **Supplementary tables:**
2. **S Table 1.** 4D flow echo-planar imaging (EPI) sequence details
3. **S Table 2.** Intra-observer and inter-observer global and regional KE parameters.
4. **S Table 3.** Non-normalised LV blood flow KE (μJ ) in controls versus MI-patients.
5. **S Table 4.** Correlation of non-normalised LV blood flow KE to infarct size.

**Tables**

**S Table 1.** 4D flow echo-planar imaging (EPI) sequence details

| Acceleration method | Parallel imaging; sense factor 2 in phase-encode AP direction  EPI factor of 5 |
| --- | --- |
| Flip-angle | 10 º |
| VENC (cm/s) | 150 |
| FOV | 350-400 |
| TE (ms) | 3.5 |
| TR (ms) | 10 |
| Partial k-space coverage in phase- encoding directions | 90% |
| Signal averages | 1 |
| ECG gating | Retrospective |
| Respiratory compensation | Free-breathing |
| Number of slices |  |
| Acquired temporal resolution (ms) | 4×TR = 40 |
| Reconstructed number of phases | 30 |
| In-plane spatial resolution (acquired) | 3×3×3mm (isotropic) |
| In-plane spatial resolution (reconstructed) | 2×2×3mm |

**S Table 2.** Intra-observer and inter-observer global KE parameters.

|  | | **Intra-observer tests** | | | | | |  | **Inter-observer tests** | | | | | |
| --- | --- | --- | --- | --- | --- | --- | --- | --- | --- | --- | --- | --- | --- | --- |
|  | | Bias (%) | LL  (%) | UL  (%) | ICC | 95% CI | P | Bias  (%) | LL  (%) | UL  (%) | ICC | 95% CI | P |  |
|  |  | **Global LV kinetic energy parameters** | | | | | | | | | | | | |
| Averaged LV | | 1 | -8 | 10 | 0.99 | 0.99 to 0.99 | 0.97 | -3 | -15 | 10 | 0.99 | 0.99 to 0.99 | 0.90 |  |
| Residual | | 5 | -23 | 32 | 0.98 | 0.94 to 0.99 | 0.86 | -6 | -33 | 21 | 0.98 | 0.94 to 0.99 | 0.79 |  |
| Systolic | | -2 | -24 | 20 | 0.99 | 0.96 to 0.99 | 0.97 | -1 | -21 | 18 | 0.99 | 0.97 to 0.99 | 0.92 |  |
| Diastolic | | 3 | -15 | 21 | 0.99 | 0.97 to 0.99 | 0.94 | -4 | -20 | 12 | 0.99 | 0.97 to 0.99 | 0.91 |  |
| Peak E-wave | | 5 | -11 | 20 | 0.99 | 0.98 to 0.99 | 0.84 | -4 | -18 | 9 | 0.99 | 0.98 to 0.99 | 0.85 |  |
| Peak A-wave | | 3 | -12 | 19 | 1.00 | 0.99 to 0.99 | 0.88 | -5 | -21 | 11 | 0.99 | 0.98 to 0.99 | 0.79 |  |
| In-plane | | -1 | -14 | 12 | 1.00 | 0.99 to 0.99 | 0.97 | 1 | -15 | 16 | 1.00 | 0.98 to 0.99 | 0.97 |  |

CI=confidence interval, LV=left ventricle, LL=lower-limit, P=p-value, UL=upper limit, ICC= inter-class correlation coefficient.

**S Table 3.** Non-normalised LV blood flow KE (μJ ) in controls versus MI-patients.

|  | Controls | | MI patients | | P-value |
| --- | --- | --- | --- | --- | --- |
|  | Median | IQR | Median | IQR |  |
| LV | 1.27 | 0.50 | 1.32 | 0.80 | 0.74 |
| Minimal | 0.14 | 0.10 | 0.13 | 0.20 | 0.84 |
| Systolic | 1.45 | 0.80 | 1.31 | 0.80 | 0.33 |
| Diastolic | 1.14 | 0.80 | 1.13 | 0.90 | 0.40 |
| Peak E-wave | 3.51 | 1.70 | 2.92 | 2.20 | 0.72 |
| Peak A-wave | 1.74 | 0.60 | 2.04 | 1.40 | 0.37 |

**S Table 4.** Correlation of non-normalised LV blood flow KE to infarct size.

|  | Correlation | P-Value |
| --- | --- | --- |
| LV | -0.01 | 0.92 |
| Minimal | 0.32 | 0.03 |
| Systolic | -0.25 | 0.09 |
| Diastolic | 0.10 | 0.49 |
| Peak E-wave | -0.09 | 0.53 |
| Peak A-wave | -0.25 | 0.09 |
